# BEYOND: a randomized controlled trial comparing efficacy and safety of individualized follitropin delta dosing in a GnRH agonist versus antagonist protocol during the first ovarian stimulation cycle

**DOI:** 10.1093/humrep/deae092

**Published:** 2024-05-09

**Authors:** Rita Lobo, Terje Soerdal, Erling Ekerhovd, Ben Cohlen, Eleonora Porcu, Michael Schenk, Yoel Shufaro, Jesper Smeenk, Moritz B Suerdieck, Philippe Pinton, Anja Pinborg, BEYOND Investigators, Omar Josef Shebl, Michael Schenk, Andreas Obruca, Anja Pinborg, Einat Shalom-Paz, Talia Eldar-Geva, Yoel Shufaro, Foad Azem, Eleonora Porcu, Ermanno Greco, Jesper Smeenk, Ben Cohlen, Terje Soerdal, Erling Ekerhovd, Peter Fedorcsak, Michael Häberle, Moritz Suerdieck, Bernadette Mannaerts, Celia J Parkyn

**Affiliations:** Clinical and Translational Sciences, Ferring Pharmaceuticals A/S, Kastrup, Denmark; Medicus AS, Trondheim, Norway; Fertility Department, Telemark Hospital, Porsgrunn, Norway; Isala Fertility Center, Zwolle, The Netherlands; Infertility and IVF Unit, IRCCS Azienda Ospedaliero, University of Bologna, Bologna, Italy; Das Kinderwunsch Institut, Dobl, Austria; Infertility and IVF Unit, Beilinson Hospital, Rabin Medical Center, Petach-Tikva, Israel; Department of Obstetrics and Gynecology, Tel-Aviv University, Tel-Aviv, Israel; Elisabeth TweeSteden Ziekenhuis, Tilburg, The Netherlands; Gyn-A.R.T. AG, Zurich, Switzerland; Clinical and Translational Sciences, Ferring Pharmaceuticals A/S, Kastrup, Denmark; Fertility Department Rigshospitalet, Copenhagen University Hospital, Copenhagen, Denmark

**Keywords:** follitropin delta, ovarian stimulation, GnRH agonist protocol, GnRH antagonist protocol, pregnancies, RCT, live births

## Abstract

**STUDY QUESTION:**

How does a gonadotrophin-releasing hormone (GnRH) agonist versus a GnRH antagonist protocol affect ovarian response when using an individualized fixed daily dose of follitropin delta for ovarian stimulation?

**SUMMARY ANSWER:**

The BEYOND trial data demonstrate thatindividualized fixed-dose follitropin delta is effective when used in a GnRH agonist protocol, compared with a GnRH antagonist protocol, in women with anti-Müllerian hormone (AMH) ≤35 pmol/l and no increased risk of ovarian hyperstimulation syndrome (OHSS).

**WHAT IS KNOWN ALREADY:**

The efficacy and safety of an individualized fixed daily dose of follitropin delta (based on body weight and AMH) have been established in randomized controlled trials (RCTs) using a GnRH antagonist protocol. Preliminary study data indicate that individualized follitropin delta is also efficacious in a GnRH agonist protocol (RAINBOW trial, NCT03564509). There are no prospective comparative data using individualized follitropin delta for ovarian stimulation in a GnRH agonist versus a GnRH antagonist protocol.

**STUDY DESIGN, SIZE, DURATION:**

This is the first randomized, controlled, open-label, multi-centre trial exploring efficacy and safety of individualized follitropin delta dosing in a GnRH agonist versus a GnRH antagonist protocol in participants undergoing their first ovarian stimulation cycle for IVF/ICSI. A total of 437 participants were randomized centrally and stratified by centre and age. The primary endpoint was the number of oocytes retrieved. Secondary endpoints included ongoing pregnancy rates, adverse drug reactions (including OHSS), live births, and neonatal outcomes.

**PARTICIPANTS/MATERIALS, SETTING, METHODS:**

Participants (18–40 years; AMH ≤35 pmol/l) were enrolled at specialist reproductive health clinics in Austria, Denmark, Israel, Italy, the Netherlands, Norway, and Switzerland. The mean number of oocytes retrieved was compared between the GnRH agonist and antagonist protocols using a negative binomial regression model with age and AMH at screening as factors. Analyses were based on all randomized subjects, using a multiple imputation method for randomized subjects withdrawing before the start of stimulation.

**MAIN RESULTS AND THE ROLE OF CHANCE:**

Of the 437 randomized subjects, 221 were randomized to the GnRH agonist, and 216 were randomized to the GnRH antagonist protocol. The participants had a mean age of 32.3 ± 4.3 years and a mean serum AMH of 16.6 ± 7.8 pmol/l. A total of 202 and 204 participants started ovarian stimulation with follitropin delta in the GnRH agonist and antagonist groups, respectively. The mean number of oocytes retrieved was statistically significantly higher in the agonist group (11.1 ± 5.9) versus the antagonist group (9.6 ± 5.5), with an estimated mean difference of 1.31 oocytes (95% CI: 0.22; 2.40, *P *=* *0.0185). The difference in number of oocytes retrieved was influenced by the patients’ age and ovarian reserve, with a greater difference observed in patients aged <35 years and in patients with high ovarian reserve (AMH >15 pmol/l). Both the GnRH agonist and antagonist groups had a similar proportion of cycle cancellations (2.0% [4/202] versus 3.4% [7/204]) and fresh blastocyst transfer cancellations (13.4% [27/202] versus 14.7% [30/204]). The estimated ongoing pregnancy rate per started cycle was numerically higher in the GnRH agonist group (36.9% versus 29.1%; difference: 7.74% [95% CI: −1.49; 16.97, *P *=* *0.1002]). The most commonly reported adverse events (≥1% in either group; headache, OHSS, nausea, pelvic pain, or discomfort and abdominal pain) were similar in both groups. The incidence of early moderate/severe OHSS was low (1.5% for the agonist group versus 2.5% for antagonist groups). Estimated live birth rates per started cycle were 35.8% and 28.7% in the GnRH agonist and antagonist groups, respectively (treatment difference 7.15%; 95% CI: −2.02; 16.31; *P *=* *0.1265). The two treatment groups were comparable with respect to neonatal health data for singletons and twins and for incidence of congenital malformations (2.7% and 3.3% for the GnRH agonist versus antagonist groups, respectively).

**LIMITATIONS, REASONS FOR CAUTION:**

All participants had AMH ≤35 pmol/l and were ≤40 years old. Clinicians should remain cautious when using a GnRH agonist protocol in patients with AMH >35 pmol/l (i.e. those with an increased OHSS risk). The incidence of OHSS in the GnRH antagonist group may have been lower if a GnRH agonist trigger had been allowed. Outcomes of transfers with cryopreserved blastocysts were not followed up, therefore the cumulative live birth rates and neonatal outcomes after cryotransfer are unknown.

**WIDER IMPLICATIONS OF THE FINDINGS:**

In women with AMH ≤35 pmol/l, an individualized fixed daily dose of follitropin delta resulted in a significantly higher number of oocytes retrieved when used in a GnRH agonist protocol compared with a GnRH antagonist protocol, with no additional safety signals observed and no additional risk of OHSS. Live birth rates following ovarian stimulation with individualized follitropin delta were not statistically different between the GnRH protocols; however, the trial was not powered to assess this endpoint. There were no safety concerns with respect to neonatal health after ovarian stimulation with follitropin delta in either protocol.

**STUDY FUNDING/COMPETING INTEREST(S):**

The trial was funded by Ferring Pharmaceuticals. EE, EP, and MS have no competing interests. AP has received research support from Ferring, and Gedeon Richter, and honoraria or consultation fees from Preglem, Novo Nordisk, Ferring, Gedeon Richter, Cryos, Merck A/S. BC has received consulting fees from Ferring and Merck, and his department received fees from Ferring to cover the costs of patient enrolment. MBS has received support to attend meetings and/or travel from Ferring, and was a board member for FertiPROTEKT e.V. until 2023. JS has received honoraria or consultation fees from Ferring and Merck, and support for attending meetings and/or travel from Ferring, Merck, and GoodLife. TS has received support/travel expenses from Ferring for attending a congress meeting, and participated in an advisory board for Merck. YS has received grants/research support from Ferring and support to attend a professional society congress meeting from Merck. RL and PP are employees of Ferring Pharmaceuticals. PP is a BOD member of PharmaBiome and owns stocks of Takeda Pharmaceuticals.

**TRIAL REGISTRATION NUMBER:**

ClinicalTrials.gov identifier NCT03809429; EudraCT Number 2017-002783-40.

**TRIAL REGISTRATION DATE:**

7 April 2019.

**DATE OF FIRST PATIENT’S ENROLMENT:**

2 May 2019.

## Introduction

GnRH agonists and antagonists are used as concomitant treatment during ovarian stimulation to prevent premature luteinization and ovulation. They share several key features, such as suppressing LH surges during ovarian stimulation, and have comparable clinical outcomes, including the number of oocytes retrieved and live birth rates (Al-Inany *et al.*, 2016; Lambalk *et al.*, 2017; Toftager *et al.*, 2017). In clinical practice, GnRH agonist protocols continue to be preferred when there is a need to schedule treatment start and ovulation induction to avoid weekend oocyte retrieval (Schultze-Mosgau *et al.*, 2005; Tremellen and Lane, 2010; Feichtinger *et al.*, 2017). The ESHRE issued international guidelines recommending the general use of GnRH antagonist protocols over GnRH agonist protocols, given the comparable efficacy and better safety profile (The ESHRE Guideline Group on Ovarian Stimulation *et al.*, 2020). This recommendation is particularly relevant for potential normal and high responders due to the risk of ovarian hyperstimulation syndrome (OHSS), whereas for poor responders, both protocols are equally recommended. Serum anti-Müllerian hormone (AMH) is one of the preferred biomarkers of ovarian response to gonadotropins. It is well-documented that there is an association between circulating AMH levels and numbers of follicles in normo-ovulatory women (Pigny *et al.*, 2003; Laven *et al.*, 2004). Potential high responders with high serum AMH may be women who are ovulatory or anovulatory, with or without polycystic ovaries (Dewailly *et al.*, 2011). They can be defined as women with AMH >35 pmol/l (Višnová *et al.*, 2021).

Follitropin delta is a commercially available recombinant follicle-stimulating hormone (rFSH) derived from a human cell line and used for ovarian stimulation in fertility patients undergoing IVF or ICSI. It was developed along with an algorithm based on body weight and AMH serum levels that determined an individualized fixed daily dose (Arce *et al.*, 2014; Follitropin Delta SmPC, 2021). The dosing algorithm aims to reduce the risk of hypo- and hyper-ovarian responses while achieving ongoing pregnancy rates comparable to those achieved with conventional FSH dosing strategies (Arce *et al.*, 2014; Olsson et al., 2014, 2015; Bosch *et al.*, 2015, 2019; Rose *et al.*, 2016; Nyboe Andersen *et al.*, 2017; Nelson *et al.*, 2019; Havelock *et al.*, 2021; Ishihara *et al.*, 2021; Qiao *et al.*, 2021).

The efficacy and safety of an individualized fixed daily dose of follitropin delta have been established in randomized control trials (RCTs) using a GnRH antagonist protocol (Nyboe Andersen *et al.*, 2017; Ishihara *et al.*, 2021; Qiao *et al.*, 2021). These Phase 3 registration trials comprised 2685 participants undergoing their first ovarian stimulation cycle as part of IVF/ICSI procedures and were conducted in Europe, North- and South America, Japan, mainland China, South Korea, Taiwan, and Vietnam. As such, the trials included a broad range of women with different ethnicities, body weights, and AMH levels. Preliminary Phase II trial data show that individualized follitropin delta may also be efficacious when used with a GnRH agonist protocol (the RAINBOW trial) (Fernández Sánchez *et al.*, 2022, 2023). However, there have been no comparative RCT data for the efficacy and safety of individualized follitropin delta in a GnRH agonist versus a GnRH antagonist protocol. As such, the objective of the exploratory BEYOND trial was to evaluate the effect of individualized fixed-dose follitropin delta treatment on ovarian response and pregnancy outcomes as part of a GnRH agonist protocol versus a GnRH antagonist protocol, including a follow-up period of 4 weeks after birth.

## Materials and methods

### Trial design

The BEYOND trial was a randomized, controlled, open-label, parallel-group, multicentre trial comparing the efficacy and safety of individualized follitropin delta dosing in first-cycle patients aged 18–40 years and undergoing ovarian stimulation for IVF/ICSI in either a long GnRH agonist protocol or a short GnRH antagonist protocol. The trial was designed to describe potential differences in the mean number of oocytes retrieved between the two GnRH analogue protocols. The trial covered one ovarian stimulation cycle and the post-trial activities covered pregnancy follow-up to live birth and 4 weeks after birth.

The BEYOND trial was performed in compliance with the International Council for Harmonisation guideline on Good Clinical Practice (GCP). Institution Ethics Committees for each study site approved the study protocol and all relevant materials used for the trial. Participants provided informed written consent. All investigators and embryologists were required to have documented GCP training completed and accredited before the start of the trial.

### Trial participants

Participants, 18–40 years old with AMH ≤35 pmol/l and BMI 17.5–32.0 kg/m^2^, were enrolled at specialist reproductive health clinics in Austria, Denmark, Israel, Italy, the Netherlands, Norway, and Switzerland and were undergoing their first IVF/ICSI cycle. The main inclusion criteria for trial participation were: good physical and mental health; tubal infertility, unexplained infertility, endometriosis stage I/II, or partners with male factor infertility; infertility for at least 1 year for participants <38 years and at least 6 months for those ≥38 years; regular menstrual cycles (24–35 days); and no strong preference for either GnRH protocol. This study defined potential high responders as women with serum AMH above 35 pmol/l (Vembu and Reddy, 2017; Višnová *et al.*, 2021). Patients were excluded from enrolling in the trial if they had: AMH >35 pmol/l (potential high responders); endometriosis stages III–IV (defined by the revised American Society of Reproductive Medicine classification); or recurrent miscarriage. All inclusion/exclusion criteria are listed in Supplementary Table S1.

### Study endpoints

The primary endpoint was the number of oocytes retrieved. The main secondary and safety endpoints were: the total follitropin delta dose and the number of stimulation days; the proportion of subjects with cycle cancellation due to poor ovarian response or excessive ovarian response; blastocyst number and quality on Day 5 after oocyte retrieval; proportion of subjects with blastocyst transfer cancellation due to risk of OHSS; pregnancy rates; frequency and intensity of adverse events; proportion of participants with either early or late OHSS of moderate/severe grade (Golan *et al.*, 1989); and the proportion of subjects with late OHSS (including OHSS of moderate/severe grade). The prespecified post-trial endpoints comprised live birth rate and neonatal health, including minor/major congenital anomalies, at birth and 4 weeks after birth.

### Randomization and treatment

Participants were randomized in a 1:1 ratio to ovarian stimulation with follitropin delta (REKOVELLE^®^ [FE 999049], Ferring Pharmaceuticals A/S, Kastrup, Denmark) in either a GnRH agonist protocol or GnRH antagonist protocol. Randomization was performed centrally through the electronic case report form (e-CRF) and was stratified by centre and according to age (<35, 35–37, and 38–40 years) using a computer-generated randomized list and was performed in blocks maintained for each trial centre. The block size of four was not disclosed to the investigators. Follitropin delta was administered as a daily subcutaneous injection in the abdomen using a fixed dose throughout the stimulation period. For participants with low AMH (<15 pmol/l), the daily follitropin delta dose was 12 μg, irrespective of body weight. For participants with high AMH (15–35 pmol/l), the daily follitropin delta dose was on a continuous scale ranging from 0.19 to 0.10 μg/kg with increasing AMH level, i.e. dependent on the participant’s AMH level and body weight.

#### Trial procedures

The GnRH agonist group received a long GnRH agonist protocol comprising subcutaneous triptorelin acetate 0.1 mg (GONAPEPTYL^®^/DECAPEPTYL^®^, Ferring Pharmaceuticals, Kastrup, Denmark) once daily, starting in the mid-luteal phase of the participants’ menstrual cycle (i.e. Days 21–24) and continued throughout the ovarian stimulation period with follitropin delta. The GnRH antagonist group received subcutaneous cetrorelix acetate 0.25 mg (Cetrotide^®^, Merck, Darmstadt, Germany) once daily, starting on stimulation Day 6 (i.e., a fixed day GnRH antagonist protocol) and continued throughout the follitropin delta stimulation period.

A single dose of human choriogonadotropin alfa 250 µg (hCGα, Ovitrelle^®^, Merck, Darmstadt, Germany) was administrated subcutaneously as soon as each participant met the criterion for triggering of final follicular maturation (≥3 follicles with a diameter ≥17 mm; if the investigator judged that ≥3 follicles of ≥17 mm could not be reached and if one or two follicles of ≥17 mm were observed, the cycle could be cancelled due to poor follicular development or triggered for final follicular maturation). Triggering with a GnRH agonist in patients allocated to the GnRH antagonist arm was not permitted to allow for a fair comparison regarding the primary endpoint of the number of oocytes retrieved.

The protocol for both treatment groups stipulated mandatory cycle cancellation if the participant had ≥25 follicles ≥12 mm at the end of stimulation, due to an increased risk of developing OHSS. This cut-off was chosen based on data from previous follitropin delta trials (Nyboe Andersen *et al.*, 2017; Ishihara *et al.*, 2021; Qiao *et al.*, 2021). Nevertheless, the investigator had the option to cancel the cycle with fewer than 25 follicles ≥12 mm if it were judged that there was a risk of OHSS. Patients who were triggered with hCG and then developed OHSS, or who were judged by the investigator to be at risk of OHSS, could either receive a blastocyst transfer or have transfer cancellation with a freeze-all strategy.

Participants <38 years at randomization had a single blastocyst transfer. Participants ≥38 years had a single blastocyst transfer if they had a good-quality blastocyst available (Grade 3BB or higher) or a double transfer if two blastocysts were available. Any remaining blastocysts could be cryopreserved and used by the participant after completion of the trial, in accordance with local guidelines and/or regulations. All procedures and assessments related to cryopreserved cycles took place outside this trial. Progesterone 100 mg vaginal tablets (Lutinus^®^, Ferring Pharmaceuticals, Kastrup, Denmark) were used three times daily, starting the day after oocyte retrieval and continued at least until the day of the βhCG test. Progesterone was terminated earlier in case of no transfer, menses, negative βhCG, or pregnancy loss.

#### Bioanalysis

Blood samples were collected at screening, before and after stimulation and evaluated by a central laboratory for measurement of serum hormones and haematology parameters, except for βhCG and blood samples for oestradiol (if applicable), which were analysed by local laboratories at the clinic or hospital. At screening, AMH, FSH, and endocrine parameters were assessed, and the results were available prior to randomization. AMH measurements were made using both Elecsys^®^ AMH Plus Immunoassay and Elecsys^®^ AMH Immunoassay (Roche Diagnostics, Basal, Switzerland). Endometrial status was assessed during ovarian stimulation and oocyte retrieval.

#### Data quality assurance and trial monitoring

An electronic case report form (e-CRF) system provided by an independent third-party contract research organization (Target Health LLC, Carlstadt, NJ, USA) was used for data capturing. Errors in the e-CRF were corrected electronically and automatically tracked by an audit trail detailing the date and time and reason of the correction and the name of the person making the correction.

General quality and consistency checks as well as medical monitoring were conducted by the sponsor throughout the trial and throughout the post-trial follow-up in accordance with the prespecified centralized monitoring plan.

### Statistical analysis

The trial sample size calculation was based on achieving a reasonable precision in estimating the difference in the mean number of oocytes retrieved with follitropin delta when used in a GnRH agonist versus a GnRH antagonist protocol for ovarian stimulation. In the ESTHER-1 trial, the standard deviation (SD) for the number of oocytes retrieved was 5.8 in the follitropin delta group (Nyboe Andersen *et al.*, 2017). As, a greater number of oocytes may be retrieved with an agonist protocol, the sample size was determined using a higher SD. Assuming an SD of 7.0, a sample size of 400 subjects (200 subjects per group) would give a 95% CI ranging from −1.4 to +1.4 of the observed mean difference.

This exploratory trial was descriptive and no formal hypothesis was tested. For the primary endpoint, the mean number of oocytes retrieved was compared between the agonist and antagonist protocols using a negative binomial regression model with age and AMH at screening as factors. The mean number of oocytes retrieved is reported for participants who received follitropin delta (safety set, as observed), as well as the estimated difference between the agonist GnRH group and the GnRH antagonist group, using a multiple imputation method for all randomized participants who discontinued prior to starting ovarian stimulation but not including participants who discontinued due to COVID-19 pandemic-related clinic closures and societal restrictions (full analysis set) to account for any potential attrition bias if there were unbalanced withdrawal rates from the two treatment arms prior to starting ovarian stimulation. For the multiple imputation analysis of the primary endpoint and live birth rates (a post-trial activity endpoint), participants who discontinued after randomization but before the start of stimulation with follitropin delta had their endpoint data imputed from participants who started stimulation with follitropin delta in the same protocol. Data were not imputed for participants who discontinued the trial after starting stimulation. The actual data were used for these participants and were not regarded as missing. Therefore, the number of oocytes retrieved was regarded as zero and pregnancy outcomes were regarded as negative. For participants who discontinued the trial after randomization but before the start of stimulation with follitropin delta, the full details of the multiple imputation method are provided in Supplementary Data File S1. The distribution of number of oocytes retrieved was described using the empirical distribution and approximated using kernel estimates. Descriptive statistics were used to describe follicular development (size and number of follicles).

The analyses of pregnancy and live birth endpoints were revised after the trial was interrupted due to the COVID pandemic to exclude participants with transfers cancelled due to clinic closures and societal restrictions relating to the pandemic, to better reflect what the pregnancy rate would have been in a normal setting (i.e. without the COVID-19 pandemic). Multiple imputation was used for randomized participants withdrawing before the start of stimulation. Differences and confidence intervals were derived using the delta method. Live birth rates were compared using a logistic regression model with age and AMH at screening as factors.

## Results

### Disposition and baseline characteristics

**Figure 1.**
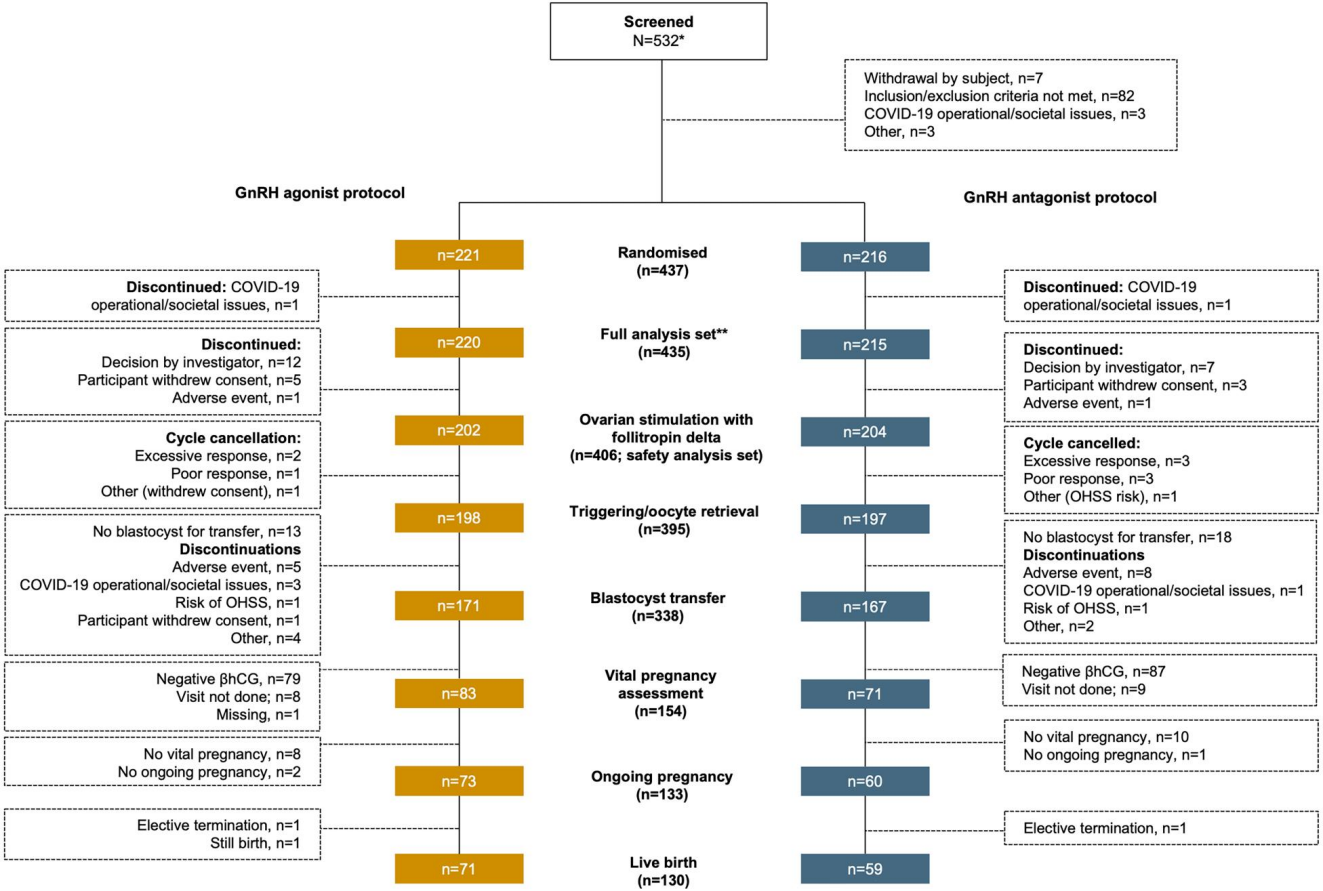
**Trial flow diagram**. *After the COVID-19 trial interruption, 10 of the 532 subjects were rescreened. **Refers to subjects who were randomized regardless of whether they started stimulation with follitropin delta or not, except the subjects who discontinued before starting stimulation with follitropin delta due to COVID-19 pandemic (e.g. due to operational and/or societal issues). βhCG, beta human chorionic gonadotropin; OHSS, ovarian hyperstimulation syndrome.

Between 2 May 2019 and 16 February 2022, 532 subjects were screened and 437 of them were randomized: 221 subjects to the GnRH agonist protocol and 216 subjects to the GnRH antagonist protocol. There were 16 trial sites across Austria, Denmark, Israel, Italy, Norway, and Switzerland. There were 202 and 204 participants who received follitropin delta as part of a GnRH agonist protocol and GnRH antagonist protocol, respectively (Fig. 1). A total of 50 participants did not complete the trial for reasons other than cycle cancellation, including 31 subjects who were randomized but did not start ovarian simulation. Reasons for discontinuation included withdrawal of consent (GnRH agonist group, n = 7; GnRH antagonist group, n = 3), adverse event (GnRH agonist group, n = 6; GnRH antagonist group, n = 9), COVID-19 pandemic societal restrictions (GnRH agonist group, n = 4; GnRH antagonist group, n = 2), or investigator’s decision (GnRH agonist group, n = 12 [spontaneous pregnancy before or during down-regulation, n = 6; ovarian cyst during down-regulation, n = 3; did not achieve down-regulation; n = 2; discovery that subject did not fulfil inclusion/exclusion criteria, n = 1]; GnRH antagonist group, n = 7 [spontaneous pregnancy, n = 4; discovery that subject did not fulfil inclusion/exclusion criteria, n = 3]).

The two treatment groups were comparable in terms of demographics and baseline characteristics.

The overall trial population had a mean age of 32.3 ± 4.3 years, body weight of 68.8 ± 11.6 kg, and baseline AMH of 16.6 ± 7.8 pmol/l (Table 1). Both groups were also similar with respect to infertility and reproductive history, menstrual cycle duration, medical history, baseline serum AMH levels, and endometrial thickness, antral follicle count, and ovarian volume. Endocrine parameters on stimulation Day 1 before first dose of follitropin delta reflected pituitary downregulation in the GnRH agonist group. Approximately half of the participants had unexplained infertility.

**Table 1.** Demographics and baseline characteristics (full analysis set).

	GnRH agonist protocol (n = 220)	GnRH antagonist protocol (n = 215)
Age, years	32.3 ± 4.4	32.4 ± 4.2
*Age category*		
<35 years	143 (65.0)	141 (65.6)
35–37 years	47 (21.4)	48 (22.3)
38–40 years	30 (13.6)	26 (12.1)
*Race*		
American Indian or Alaska native	0	2 (0.9)
Asian	9 (4.1)	8 (3.7)
Black or African American	3 (1.4)	2 (0.9)
Native Hawaiian/Pacific islander	0	1 (0.5)
White	208 (94.5)	202 (94.0)
Bodyweight, kg	68.7 ± 11.6	68.6 ± 11.6
BMI, kg/m^2^	24.4 ± 3.5	24.4 ± 3.6
*Primary reason for infertility*		
Unexplained infertility	113 (51.4)	108 (50.2)
Tubal factor	20 (9.1)	26 (12.1)
Male factor	84 (38.2)	80 (37.2)
Endometriosis stage I/II	2 (0.9)	1 (0.5)
Other	1 (0.5)	0
Primary infertility	141 (64.1)	152 (70.7)
Duration of infertility, months	31.2 ± 25.0	29.5 ± 21.1
Endometrial thickness, mm	2.7 ± 1.0	3.5 ± 1.5
Ovarian volume, cm^2^	5.1 ± 3.4	5.7 ± 2.7
Antral follicle count	16.1 ± 6.6	15.0 ± 5.8
AMH at screening, pmol/l	16.9 ± 7.5	16.3 ± 8.1
*AMH category*		
<15 pmol/l	92 (41.8)	102 (47.4)
≥15–35 pmol/l	128 (58.2)	113 (52.6)
≥25–35 pmol/l	36 (16.4)	39 (18.1)
Follicle stimulation hormone at screening, IU/l	8.1 ± 2.0	8.1 ± 2.0
*Endocrine parameters on stimulation Day 1^a^*	n = 202	n = 204
Follicle stimulation hormone, IU/l	4.1 (3.2–5.2)	8.0 (6.8–9.5)
LH, IU/l	2.2 (1.6–3.4)	4.8 (6.8–9.5)
Oestradiol, pmol/l	34.9 (34.9–89.6)	155.6 (121.3–198.9)
Progesterone, nmol/l	0.8 (0.8–2.0)	1.9 (0.8–2.9)
Inhibin B, pg/ml	13.0 (5.0–25.0)	84.4 (58.0–113.0)

a Endocrine parameters were assessed for all participants who started ovarian stimulation prior to first dose of follitropin delta; participants in the GnRH agonist group were down-regulated. Participants in the GnRH agonist group started the GnRH agonist treatment on Days 21–24 of the participant’s menstrual cycle and was continued throughout the stimulation period.

Data are mean±SD, median (interquartile range), or n (%). AMH, anti-Müllerian.

### Use of noninvestigational drugs

The GnRH agonist was administered for a mean ± SD of 25.3 ± 2.8 days starting on Days 21 to 24 of the participant’s menstrual cycle and was continued throughout the stimulation period. The GnRH antagonist was administered for 4.2 ± 1.7 days starting on stimulation Day 6 and was continued throughout the stimulation period.

Triggering of final follicular maturation was conducted for 198 and 197 participants in the agonist and antagonist groups, respectively. Among the participants who underwent triggering in the GnRH agonist group, 194 met the triggering criterion, three had one or two follicles with a diameter ≥17 mm, and one had 25 follicles ≥12 mm and underwent triggering despite meeting the criteria for cycle cancellation. Among the participants who underwent triggering in the antagonist group, 191 met the triggering criterion and six had one or two follicles with a diameter ≥17 mm.

Overall, participants used similar types of concomitant medications in both GnRH protocol groups. The most frequently used concomitant medications were anaesthetics (377/435 [86.7%]), analgesics (344/435 [79.1%]), psycholeptics (259/435 [59.5%]), anti-inflammatory and antirheumatic drugs (217/435 [49.9%]), and anti-anaemic medications (162/435 [37.2%]), the use of which were mostly related to oocyte retrieval and blastocyst transfer procedures.

### Ovarian stimulation

Follitropin delta dosing for ovarian stimulation is summarized in Table 2. Participants in the GnRH agonist group had a longer mean duration of stimulation and a higher mean total dose used. Both GnRH analogue protocol groups had similar mean fixed daily doses. Dose adjustments during stimulation were not permitted.

**Table 2.** Ovarian stimulation, fertilization, and blastocyst transfer (safety analysis set).

	GnRH agonist protocol (as observed, n = 202)	GnRH antagonist protocol (as observed, n = 204)	Estimated difference (95% CI) and *P-value*
*Follitropin delta dosing*			
Duration, days	10.4 ± 1.9	8.8 ± 1.8	–
Daily dose, µg	10.8 ± 1.7	10.9 ± 1.8	–
Total dose, µg	112.2 ± 28.9	96.5 ± 26.0	–
Oocytes retrieved (primary endpoint)	11.1 ± 5.9	9.6 ± 5.5	1.31 (0.22; 2.40);***P = 0.0185***^a^
*Ovarian response stratified by AMH*			
Participants with AMH <15 pmol/l (at risk of hypo-response)	n = 84	n = 97	–
Oocytes retrieved	8.8 ± 5.0	8.0 ± 4.9	0.88 (−0.53; 2.28); *P = 0.2217^b^*
<4 oocytes	9 (10.7)	15 (15.5)	–
<8 oocytes	38 (45.2)	52 (53.6)	–
Participants with AMH 15–35 pmol/l (at risk of hyper-response)	n = 118	n = 107	–
Oocytes retrieved	12.8 ± 6.0	11.0 ± 5.7	1.73 (0.09; 3.36) ***P = 0.0383****^b^*
≥15 oocytes	47 (36.7)	30 (26.5)	–
≥20 oocytes	13 (11.0)	9 (8.4)	–
*Cycle cancellation*	4 (2.0)	7 (3.4)	–
Excessive response	2 (1.0)	3 (1.5)	–
Poor response	1 (0.5)	3 (1.5)	–
Other reason	1 (0.5)	1 (0.5)	–
*Method of fertilization*			
ICSI	96 (47.5)	99 (48.5)	–
IVF	85 (42.1)	89 (43.6)	–
ICSI and IVF	14 (6.9)	7 (3.4)	–
No fertilization	7 (3.5)	9 (4.4)	–
Metaphase II oocytes^c^	9.2 ± 4.9	7.9 ± 4.7	1.11 (−0.20; 2.41); *P = 0.0962^c^*
Fertilized oocytes	5.9 ± 4.0	5.2 ± 3.9	0.48 (−0.30; 1.25) *P = 0.2282^a^*
*Blastocysts on Day 5 after fertilization*			
Blastocysts	3.8 ± 3.1	3.3 ± 2.9	0.37 (−0.20; 0.94) *P = 0.202^a^*
Good-quality blastocysts^d^	2.3 ± 2.3	2.1 ± 2.2	0.12 (−0.31; 0.54) *P = 0.5946^a^*
Blastocyst transfer	171 (84.7)	167 (81.9)	–
Blastocyst transfer cancellation	27 (13.4)	30 (14.7)	–
		13 (6.4)	
*Primary reason for transfer cancellation*			
No blastocyst	13 (6.4)	13 (6.4)	–
Adverse event	5 (2.5)	8 (3.9)	–
COVID-19	3 (1.5)	1 (0.5)	–
Risk of OHSS	1 (0.5)	1 (0.5)	–
Other reason	5 (2.5)	2 (1.0)	–

Data are mean ± SD, median (interquartile range), or n (%) unless stated otherwise. *P*-values in bold text are less than 0.05 (i.e., statistically significant).

a Endpoint comparative analysis was based on all randomized subjects, except those withdrawing before start of stimulation due to COVID-19 pandemic societal restrictions, with multiple imputations that used a negative binomial model adjusted for age strata and AMH group at baseline (full analysis set; GnRH agonist group: n = 220; GnRH antagonist group: n = 215).

b Treatment group comparative analyses by subgroup were based on all randomized subjects using a multiple imputation method for subjects withdrawing before the start of stimulation (GnRH agonist group: AMH <15 pmol/l, n = 92; AMH 15–35 pmol/l, n = 128; GnRH antagonist group: AMH <15 pmol/l, n = 102; AMH 15–35 pmol/l, n = 113).

c Metaphase oocytes were assessed for participants who had all oocytes fertilized by ICSI (GnRH agonist group: n = 96; GnRH antagonist group: n = 99).

d A good-quality blastocyst was defined as a blastocyst of Grade 3BB or higher.

OHSS, ovarian hyperstimulation syndrome.

#### Ovarian response

**Figure 2.**
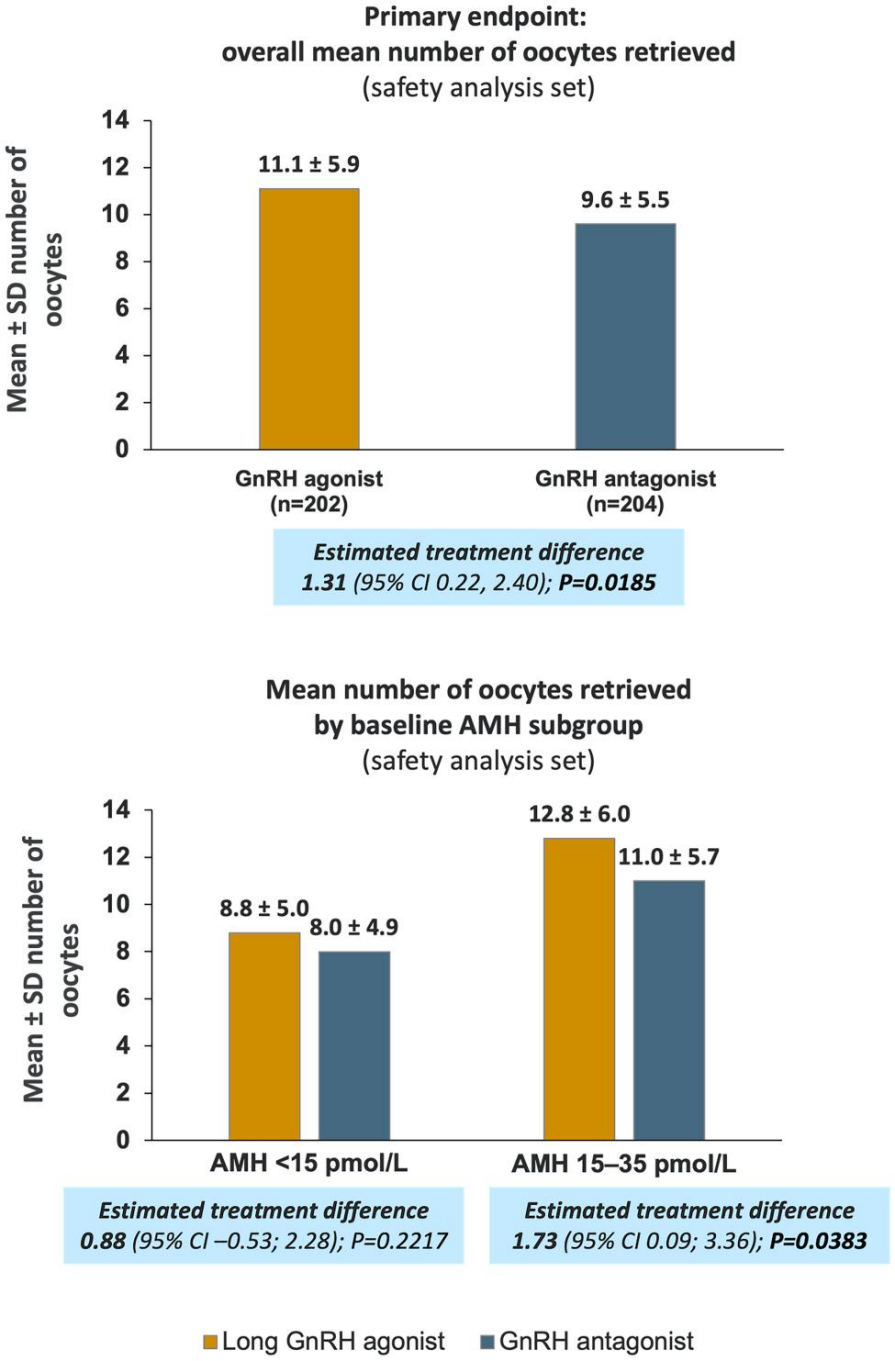
**Number of oocytes retrieved (ovarian response)**. Safety analysis set consists of all subjects who were randomized and exposed to treatment, i.e. subjects who started ovarian stimulation (GnRH agonist group: n = 202; GnRH antagonist group: n = 204). Comparative analyses (estimated treatment differences) were calculated using the full analysis set with multiple imputation for the subjects who did not start ovarian stimulation derived using a negative binomial regression model with treatment, age stratum, and AMH at screening as factors (GnRH agonist group: n = 220; GnRH antagonist group: n = 215). AMH, anti-Müllerian hormone.

The mean number of oocytes retrieved among participants who started ovarian stimulation was 11.1 and 9.6 in the GnRH agonist and antagonist treatment groups, respectively (Table 2 and Fig. 2). For the primary analysis using multiple imputations for participants who did not start ovarian stimulation, the estimated difference was 1.31 (95% CI: 0.22; 2.40; *P *=* *0.0185). Comparable results were obtained when the analysis was repeated for just the participants who underwent ovarian stimulation without using multiple imputations (as observed; data not shown).

**Figure 3.**
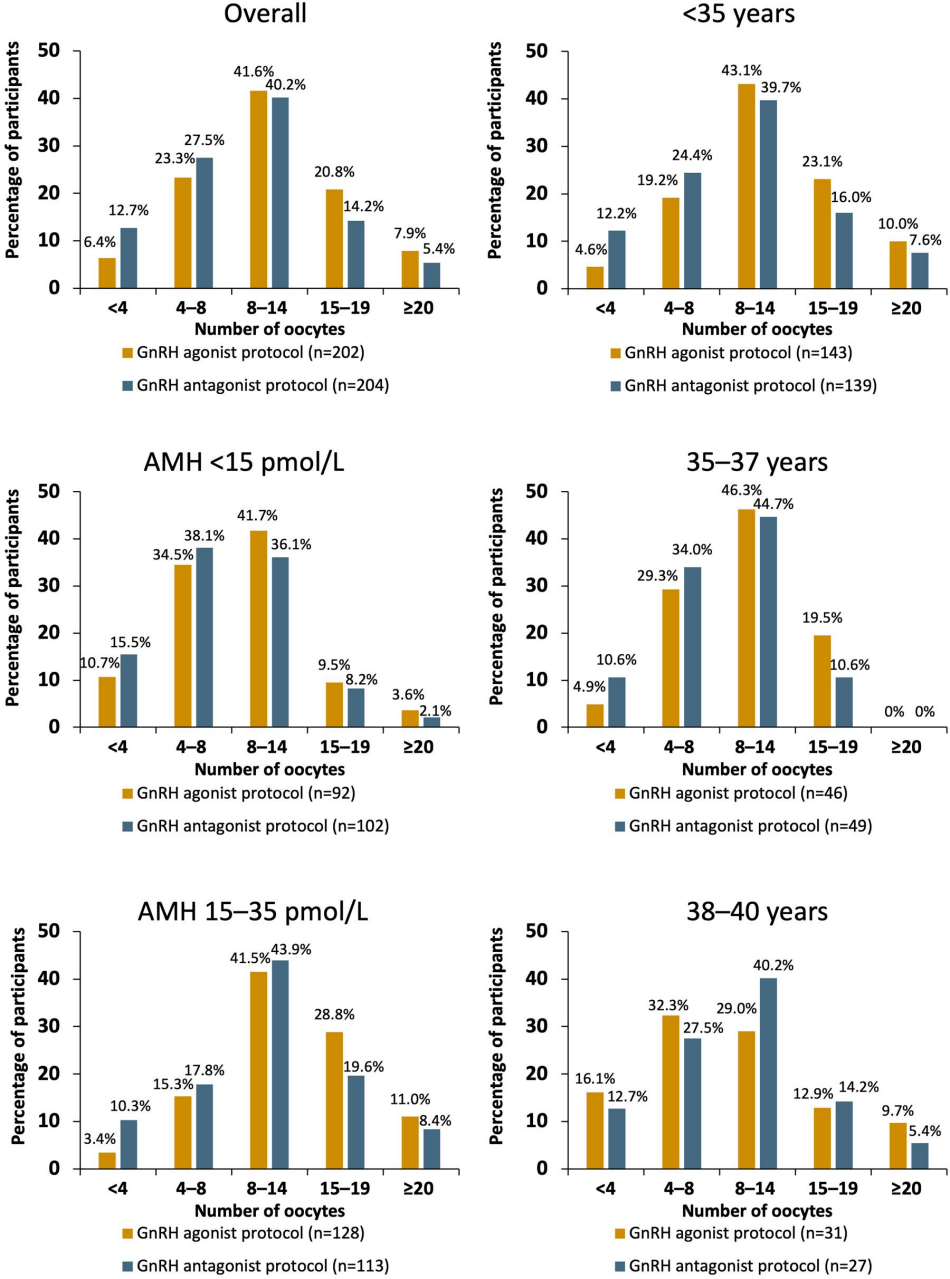
**Ovarian response categorized by number of oocytes retrieved: overall and AMH and age subgroups (safety analysis set)**. AMH, anti-Müllerian hormone.

The category of 8–14 oocytes was the most common ovarian response in both the agonist and the antagonist protocol groups overall and for all AMH and age subgroups (Fig. 3).

When analysed by AMH subgroup, the mean number of oocytes retrieved was lower among participants with low AMH levels (<15 pmol/l: 8.8 ± 5.0 and 8.0 ± 4.9) compared with high AMH levels (15–35 pmol/l: 12.8 ± 6.0 and 11.0 ± 5.7) for GnRH agonist and antagonist protocol groups, respectively (Fig. 2). For participants with low AMH (<15 pmol/l), the estimated mean difference in the number of oocytes retrieved was 0.88 [95% CI: −0.53; 2.28] for the two protocols, which was not significantly different (*P *=* *0.2217; multiple imputation analysis). For participants with high AMH (15–35 pmol/l), the estimated mean difference in the number of oocytes was 1.73 [95% CI: 0.09; 3.36], which was significantly higher in the GnRH agonist protocol compared with the GnRH antagonist protocol (*P *=* *0.0383; multiple imputation analysis) (Fig. 2).

When analysed by age subgroups, a greater number of oocytes were retrieved for participants <35 years who received the GnRH agonist protocol (estimated treatment difference 1.52 [95% CI: 0.05; 2.99]; *P *=* *0.0431). There were no significant differences between the two protocols for participants 35–37 or 38–40 years (Supplementary Table S2).

### Endocrine and endometrial parameters

Serum progesterone and oestradiol levels are summarized in Table 3. There was no significant difference between the two GnRH treatment groups in progesterone levels at the end of stimulation with follitropin delta or on the day of oocyte retrieval. Although there was a significant difference between the two GnRH protocol groups in serum oestradiol levels at the end of stimulation with follitropin delta, the difference was not statistically or clinically significant on the day of oocyte retrieval.

**Table 3.** Endocrine parameters in participants who received follitropin delta (safety analysis set).

Mean	GnRH agonist protocol	GnRH antagonist protocol
	n = 202	n = 204
Oestradiol, pmol/l		
Stimulation Day 1	34.9 (34.9–89.6)	155.6 (121.3–198.9
Stimulation Day 6	899.5 (438.1–1482.7)	2408.7 (1426.8–3678.1)
End of stimulation	6846.6 (4794.7–9584.7)	5645.9 (3782.9–8023.6)
Oocyte retrieval	3174.1 (2041.6–4471.2)	2861.9 (1766.3–4091.2)
Progesterone, nmol/l		
Stimulation Day 1	0.8 (0.8–2.0)	1.9 (0.8–2.9)
Stimulation Day 6	1.8 (0.8–2.4)	2.4 (1.7–3.4)
End of stimulation	2.8 (2.1–3.9)	2.9 (2.3–4.3)
Oocyte retrieval	23.9 (16.3–32.6)	23.8 (17.1–32.3)

Data are median (interquartile range).

Progesterone values below LLOQ (<1.59 nmol/l) are set to LLOQ/2, i.e. 0.79 nmol/l rounded off to 0.8 nmol/l. Oestradiol values below LLOQ (<69.8 pmol/l) are set to LLOQ/2, i.e. 34.9 pmol/l.

LLOQ, lower limit of quantification.

The endometrial status (endometrial thickness, endometrial triple-layer structure, and endometrial echogenicity pattern) was comparable between the two protocols on stimulation Day 6 as well as at end-of-stimulation (data not shown).

### Cycle cancellations

For participants who started stimulation with follitropin delta, cycle cancellation occurred for 4/220 participants (2.0%) in the agonist group and 7/215 subjects (3.4%) in the antagonist group. Reasons for cycle cancellation for the two groups, respectively, were excessive ovarian response: n = 2 (1.0%) and n = 3 (1.5%); poor ovarian response: n = 1 (0.5%) and n = 3 (1.5%); withdrew from trial: n = 1 (0.5%; agonist group); risk of OHSS: n = 1 (0.5%; antagonist group).

### Blastocyst quality and transfer

A similar proportion of participants who started ovarian stimulation with follitropin delta had a blastocyst transfer: 84.7% (171/202) in the GnRH agonist protocol (169 with single blastocyst transfer and two with double blastocyst transfer) and 81.9% (167/204) in the GnRH antagonist protocol (all with a single blastocyst transfer). The number of good-quality blastocysts was similar in both GnRH protocol groups (Table 2).

### Safety outcomes

**Figure 4.**
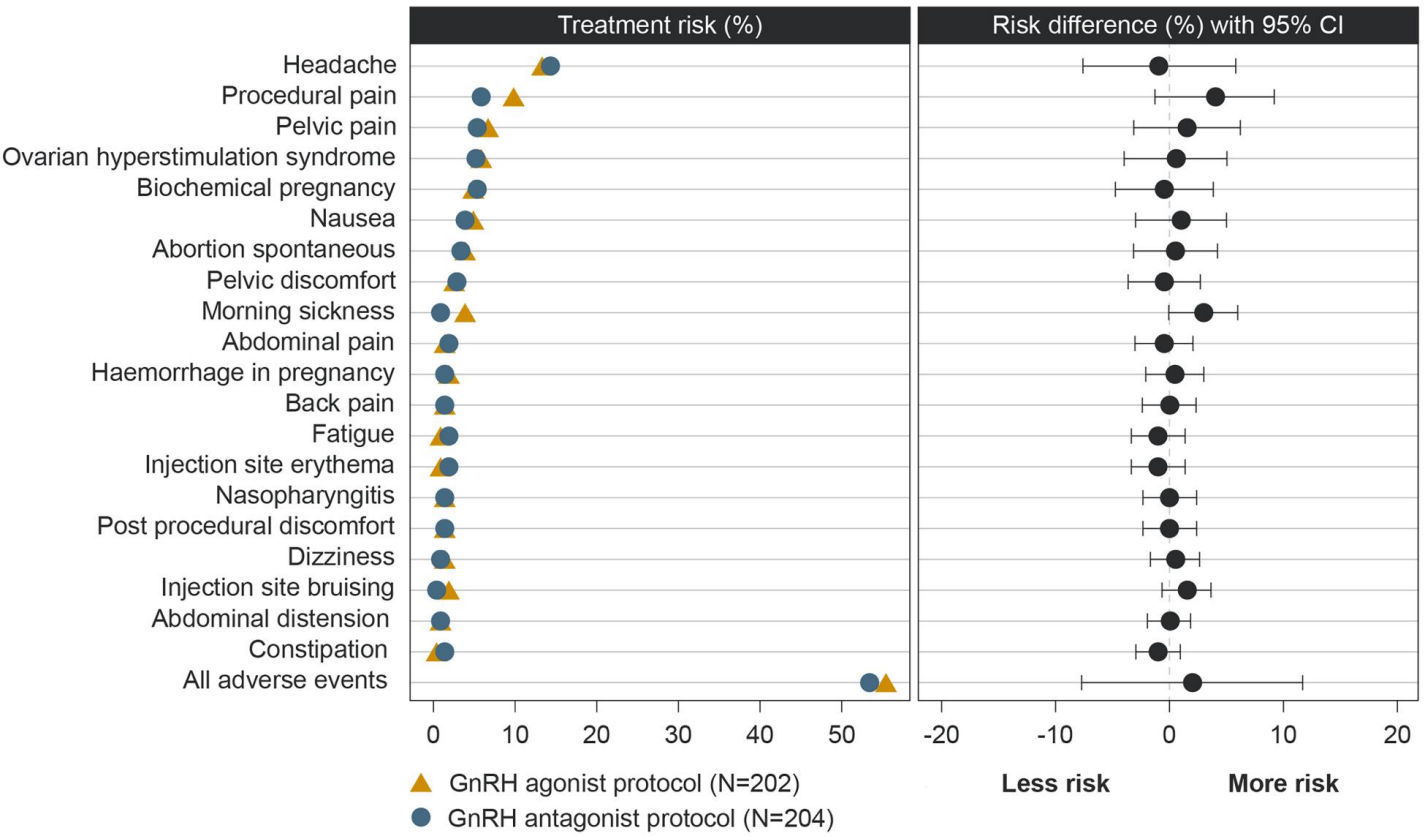
**The most frequent treatment-emergent adverse events for participants who started ovarian stimulation with follitropin delta (safety analysis set)**.

The overall proportions of participants experiencing adverse events and serious adverse events were similar in both the agonist and antagonist treatment groups (Table 4 and Fig. 4). A total of 13 participants discontinued from the trial due to an adverse event, comprising 5/202 participants (2.5%) in the GnRH agonist group (COVID-19 infection, n = 1; OHSS, n = 4) and 8/204 participants (3.9%) in the antagonist group (increased progesterone, n = 1; adnexal torsion, n = 1; endometrial disorder, n = 1; hydrometra, n = 1; uterine polyp, n = 1). All AEs leading to discontinuation occurred after completion of ovarian stimulation but resulted in fresh blastocyst transfer cancellation.

**Table 4.** Overview of adverse events in participants who received follitropin delta (safety analysis set).

	GnRH agonist protocol	GnRH antagonist protocol
	n = 202	n = 204
Adverse events	112 (55.4)	109 (53.4)
Serious adverse events	4 (2.0)	6 (2.9)
Adverse events leading to discontinuation	5 (2.5)	8 (3.9)
Severe adverse events	8 (4.0)	7 (3.4)
Adverse drug reactions	39 (19.3)	34 (16.7)
Adverse events leading to death	0	0
*Adverse events occurring in ≥1% in either study group*		
Headache	27 (13.4)	29 (14.2)
Procedural pain	20 (9.9)	12 (5.9)
Pelvic pain	14 (6.9)	11 (5.4)
OHSS (any grade)	12 (5.9)	11 (5.4)
Early OHSS (onset ≤9 days after triggering)	8 (4.0)	5 (2.5)
Late OHSS (onset >9 days after triggering)	4 (2.0)	6 (2.9)
OHSS (moderate/severe; Grades 3–5)	6 (3.0)	11 (5.4)
Early OHSS (onset ≤9 days after triggering)	3 (1.5)	5 (2.5)
Late OHSS (onset >9 days after triggering)	3 (1.5)	6 (2.9)
Biochemical pregnancy	10 (5.0)	11 (5.4)
Nausea	10 (5.0)	8 (3.9)
Spontaneous abortion	8 (4.0)	7 (3.4)
Morning sickness	8 (4.0)	2 (1.0)
Injection site bruising	4 (2.0)	1 (0.5)
Haemorrhage in pregnancy	4 (2.0)	3 (1.5)
Abdominal pain	3 (1.5)	4 (2.0)
Dizziness	3 (1.5)	2 (1.0)
Back pain	3 (1.5)	3 (1.5)
Nasopharyngitis	3 (1.5)	3 (1.5)
Postprocedural discomfort	3 (1.5)	3 (1.5)
Injection site erythema	2 (1.0)	4 (2.0)
Fatigue	2 (1.0)	4 (2.0)
Injection site rash	0	3 (1.5)
Constipation	1 (0.5)	3 (1.5)

OHSS, ovarian hyperstimulation syndrome.

#### Ovarian hyperstimulation syndrome

The incidence of OHSS of any grade was similar in both the agonist and the antagonist groups (12/202 [5.9%] and 11/204 [5.4%], respectively). There were three (1.5%) and five (2.5%) cases of moderate/severe (Grades 3–5) early OHSS with an onset ≤9 days after triggering of final follicular maturation, respectively (Table 4). The other cases were all late OHSS. OHSS was the most common adverse event leading to discontinuation (agonist group, n = 4; antagonist group, n = 3).

### Ongoing pregnancies and post-trial outcomes

Pregnancies, live births, and neonatal outcomes are summarized in Table 5.

**Table 5.** Pregnancy, live birth, and neonatal outcomes (safety analysis set).

	GnRH agonist protocol	GnRH antagonist protocol	Estimated difference (95% CI) and *P-value*
	n = 202	n = 204	
Positive βhCG test	91 (45.0)	79 (38.7)	6.99 (−2.71; 16.70*)*
			*P = 0.1579^a^*
*Ongoing pregnancies*			
Ongoing pregnancy (all participants) per started cycle (multiple imputations)	73 (36.1)	60 (29.4)	7.74 (−1.49; 16.97)
			*P = 0.1002^a^*
Singleton	69	60	*–*
Twins	4	1	
Ongoing pregnancy per started cycle in low AMH (<15 pmol/l) subgroup	31 (36.9)	32 (33.0)	_
	[n = 92]	[n = 102]	
Ongoing pregnancy per started cycle in high AMH (15–35 pmol/l) subgroup	42 (35.6)	28 (26.2)	_
	[n = 128]	[n = 113]	
Ongoing pregnancy rate per transfer	42.7%	35.9%	–
Participants with live birth (per started cycle)	71 (35.1)	59 (28.9)	7.15 (−2.02; 16.31)
			*P = 0.1265^a^*
Participants with live neonate(s) at 4 weeks after birth (per started cycle)	70 (34.7)	59 (28.9)	6.59 (−2.56; 15.73)
			*P = 0.1580^a^*
Number of live neonates at birth, n	75	60	–
Singletons, n	67	58	–
Twins, n	8	2	–
Live neonates at 4 weeks, n	73	60	–
Singletons, n	67	58	–
Twins, n	6^b^	2	–
***Neonatal outcomes at birth***			
Gestational age, weeks	38.9 ± 3.02	39.6 ± 1.81	–
Birthweight, g	3294 ± 740	3376 ± 607	–
*Apgar scores*			
1 min	8.4 ± 1.5	8.6 ± 1.4	–
5 min	9.5 ± 0.7	9.4 ± 1.1	–
10 min	9.8 ± 0.5	9.7 ±0.7	–
Admissions to NICU	10 (13.3)	6 (10.0)	–
*Congenital malformation*	2 (2.7)	2 (3.3)	–
Heart malformation	1 (1.3)	1 (1.7)	–
Foot malformation	1 (1.3)	1 (1.7)	–

a Primary analysis was based on all randomized subjects using a multiple imputation method for randomized subjects withdrawing before start of stimulation but did not include subjects who had cycle cancellation due to logistical reasons related to the COVID-19 pandemic (GnRH agonist group: n = 217; GnRH antagonist group: n = 214).

b There were two neonatal deaths in the GnRH agonist group—a set of monochorionic twins born at gestational age 24 weeks+2 days died shortly after birth due to prematurity.

Data are mean±SD or n (%) unless stated otherwise.

AMH, anti-Müllerian hormone; NICU, neonatal intensive care unit.

#### Pregnancies and live births

Among the participants who started ovarian stimulation, the positive βhCG rate was 45.0% (91/202) and 38.7% (79/204) for the agonist and antagonist groups, respectively, and the ongoing pregnancy rate was 36.1% (73/202) and 29.4% (60/204), respectively (neither were significantly different). Of the participants who had a positive βhCG test, 19.8% (18/91) in the agonist group and 24.1% (19/79) in the antagonist group experienced early pregnancy loss. There was no significant difference in early pregnancy loss rates between the two groups.

Based on the full analysis set, the estimated ongoing pregnancy rate was 36.9% and 29.1% in the GnRH agonist an antagonist groups, respectively (multiple imputation; estimated mean difference: 7.7% [95% CI: −1.49; 16.97]; *P *=* *0.1002). There was a numerically greater number of ongoing pregnancies in the agonist group (35.6% [42/128]) versus the antagonist group (26.2% [28/113]) for participants with AMH 15–35 pmol/l. There was also a numerically greater number of ongoing pregnancies in the agonist group (40.8% [53/143]) versus the antagonist group (30.5% [40/139]) for participants <35 years.

The post-trial follow-up analysis included all 133 participants with ongoing pregnancies (138 foetuses). There were three late pregnancy losses (agonist group, n = 2; antagonist group, n = 1). These comprised two elective terminations due to foetal congenital malformation (n = 1 in each treatment group) and one stillbirth at 39 weeks + 1 day for a participant in the agonist group who had cholestasis.

There were 130 live births (agonist group: 75 neonates [67 singletons; four sets of twins]; antagonist group: 60 neonates [58 singletons; one set of twins]). The estimated live birth rates per started cycle were 35.8% and 28.7% in the agonist and antagonist groups, respectively (estimated mean treatment difference 7.15%; 95% CI: −2.02; 16.31; *P *=* *0.1265). For twin live births, four participants in the agonist group had twin live births (three after a single blastocyst transfer and one after a double blastocyst transfer), and one participant in the antagonist group had a twin live birth (after a single blastocyst transfer).

#### Neonatal outcomes

There were 135 live-born neonates: 75 in the GnRH agonist protocol group and 60 in the antagonist group. The two treatment groups were comparable with respect to neonatal health data for singletons and twins and incidence of congenital malformations (2/75 [2.7%] and 2/60 [3.3%], for the agonist and antagonist groups, respectively).

There were no neonatal deaths reported within the first 24 h of birth; however, there were two neonatal deaths from the agonist group (a set of monochorionic twin pregnancy) who were born prematurely at 24 weeks + 2 days of gestation via caesarean section due to general inflammatory signs. Both neonates were admitted to the neonatal intensive care unit at birth due to prematurity and died at 3 and 4 days after birth, respectively, and these deaths were assessed as not related to the treatment protocol. Neonatal admissions to intensive care units were similar for both groups (10 and 6 neonates, respectively, from the agonist and antagonist groups).

## Discussion

**Figure 5.**
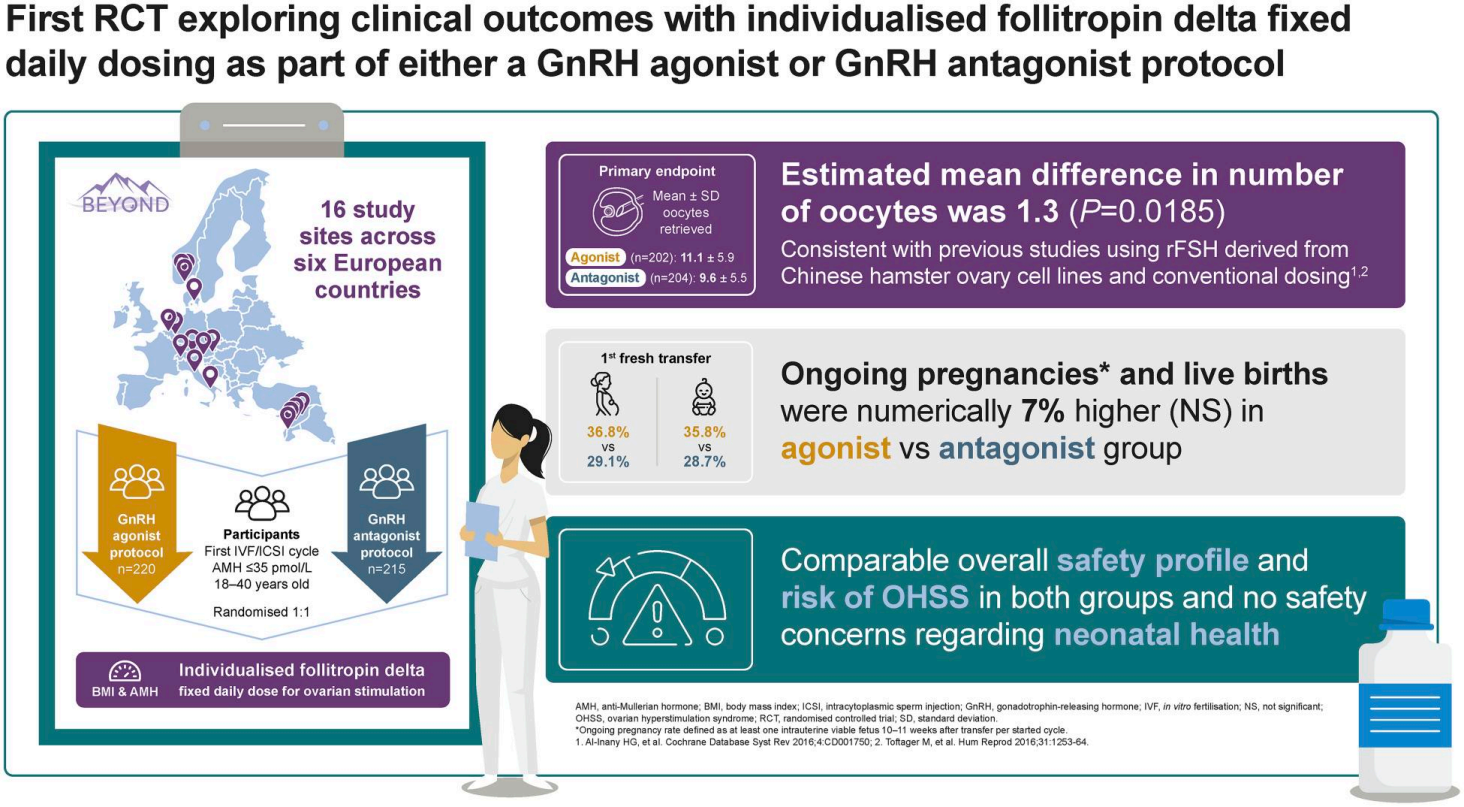
**Summary of the BEYOND trial**.

The BEYOND trial is the first prospective RCT to compare individualized follitropin delta in either a GnRH agonist or GnRH antagonist protocol for ovarian stimulation in women with AMH ≤35 pmol/l (Fig. 5). Overall, this exploratory trial demonstrates an estimated mean difference of 1.3 extra oocytes retrieved for the agonist group compared with the antagonist group (95% CI: 0.22; 2.40; *P *=* *0.0185). Moreover, greater differences were found in the subgroup of younger women (<35 years), for whom the estimated mean difference was 1.5 extra oocytes retrieved; 95% CI: 0.05; 2.99; *P *=* *0.0431), and in women with normal/high ovarian reserve (AMH 15–35 pmol), for whom the estimated mean difference of 1.7 extra oocytes retrieved (95% CI: 0.09; 3.36; *P *=* *0.0383). These results are in accordance with results from previous assisted reproduction technology studies that used conventionally dosed rFSH derived from Chinese hamster ovary cell lines in GnRH agonist and antagonist protocols (Al-Inany *et al.*, 2016; Toftager *et al.*, 2017).

In the BEYOND trial, the adverse events reported were similar in both the GnRH agonist and antagonist treatment groups. As such, the safety profile of the GnRH agonist protocol appears to be improved with follitropin delta compared with previously reported trials that used conventionally dosed rFSH. This finding is surprising since it is known that GnRH agonist and antagonist protocols sometimes display different safety profiles with conventional rFSH dosing (Cetrotide SmPC, 2009; Gonapeptyl Monograph, 2019). For example, GnRH agonist treatment is associated with adverse drug reactions related to hypoestrogenism (mild to severe hot flushes and vaginal dryness), ovarian cysts during the initial phase of treatment, and a higher incidence of OHSS, compared with treatment with a GnRH antagonist protocol, especially in predicted normal to high responders (Al-Inany *et al.*, 2016; Lambalk *et al.*, 2017; Toftager *et al.*, 2017; Yang *et al.*, 2021). In the BEYOND trial, adverse events related to hypoestrogenism or flare-ups were not observed. Importantly, the incidences of total OHSS and early moderate/severe OHSS were similar between the two groups. These findings are reassuring as they suggest that women with AMH ≤35 pmol/l are unlikely to be at higher risk of an excessive ovarian response if they receive individualized fixed-dose follitropin delta as part of a GnRH agonist protocol compared with a GnRH antagonist protocol. Moreover, there were no neonatal health concerns after ovarian stimulation with follitropin delta in either GnRH protocol.

The BEYOND trial showed a numerically (∼7%) higher pregnancy rate and live birth rate in the GnRH agonist group. Although there were no statistically significant differences between the two protocols regarding the number and quality of blastocysts or pregnancy and live birth rates, the trial was not powered to detect differences in these outcomes, and therefore, these results should be interpreted with caution. Moreover, there were no statistically significant differences in circulating oestradiol or progesterone levels, or endometrial status on the day of oocyte retrieval between the two treatment groups. Previous studies described contrasting findings regarding ongoing pregnancy rates and live birth rates. Some authors have reported no differences in ongoing pregnancy rates (Al-Inany *et al.*, 2016; Toftager *et al.*, 2017). Conversely, others describe higher pregnancy rates related to treatment with GnRH agonists (Lambalk *et al.*, 2017). For live birth rates, previous studies showed that there are no statistically significant or conclusive evidence of differences between the two GnRH analogue protocols (Al-Inany *et al.*, 2016; Lambalk *et al.*, 2017; Toftager *et al.*, 2017). More research is needed to better understand whether the higher pregnancy and live birth rates observed in the GnRH agonist group were due to mere chance in the BEYOND trial. Such trials would require a larger sample size to be powered to detect any potential differences in ongoing pregnancy and live birth rates.

Although the BEYOND trial was based in Europe, the efficacy and safety data of follitropin delta when used in a GnRH agonist protocol may be applicable to non-European populations, as there are strong similarities between the efficacy and safety in the antagonist-treatment arm of this trial and previous follitropin delta phase 3 trials that used GnRH antagonist protocols in Europe and rest of world (ESTHER-1 trial), and Asia (STORK and GRAPE trials) (Nyboe Andersen *et al.*, 2017; Ishihara *et al.*, 2021; Qiao *et al.*, 2021). The BEYOND and ESTHER-1 trial populations had very similar baseline patient characteristics, whereas the Asian trial populations were marginally younger, had a lower mean body weight and slightly higher baseline AMH. The mean number of oocytes retrieved (9.6 oocytes) in the BEYOND antagonist group was similar to the ESTHER-1 and GRAPE trials (10.0 oocytes for both trials in the follitropin delta arms) and slightly higher than the Japanese population in the STORK trial (7.2 oocytes) (Nyboe Andersen *et al.*, 2017; Ishihara *et al.*, 2021; Qiao *et al.*, 2021). Live birth rates following ovarian stimulation with individualized follitropin delta and fresh transfer were: 29.8% for ESTHER, 23.5% for STORK, and 31.3% for GRAPE (Nyboe Andersen *et al.*, 2017; Ishihara *et al.*, 2021; Qiao *et al.*, 2021).

Furthermore, the results of the BEYOND trial corroborate the preliminary findings from the phase 2 RAINBOW trial in which individualized follitropin delta was used with a GnRH agonist protocol (Fernández Sánchez *et al.*, 2022, 2023). In the RAINBOW trial, the follitropin delta group had a mean of 12.5 oocytes retrieved, (compared with a mean of 11.1 oocytes retrieved in the BEYOND trial’s agonist group) and live birth rate of 43% after fresh transfer (Fernández Sánchez *et al.*, 2022, 2023).

It is important to highlight that in the BEYOND trial, women with AMH >35 pmol/l (potential high responders) were excluded since it is known that the agonist protocol increases the risk of OHSS, especially for potential high responders (The ESHRE Guideline Group on Ovarian Stimulation *et al.*, 2020). In contrast, there were no restrictions regarding AMH levels in the phase 3 follitropin delta trials using a GnRH antagonist protocol. Nevertheless, there were more cycle cancellations due to excessive ovarian response and a higher incidence of OHSS in the antagonist group in the BEYOND trial compared with the ESTHER-1, STORK, and GRAPE trials, in part due to the stipulated cycle cancellation being ≥25 follicles ≥12 mm (compared with 35 follicles ≥12 mm in the other trials) and because using a GnRH agonist for triggering was not allowed in the antagonist group in the BEYOND trial, but could be used in the other trials if ≥25 follicles ≥12 mm were observed at the end of stimulation or if it were judged by the investigator that there was a risk of OHSS. In the BEYOND trial, OHSS incidence in the GnRH antagonist group may have been lower if a GnRH agonist trigger had been allowed; however, it would have confounded the interpretation of any potential differences in ovarian response among the two groups. Currently, it is standard practice to use a GnRH antagonist protocol with a GnRH agonist trigger and a ‘freeze-all’ strategy in accordance with strict criteria. The antagonist protocol also reduces the length and complexity of the treatment cycle, thereby reducing the physical and psychosocial burden on the patient (Toftager *et al.*, 2018).

When mitigating the risk of OHSS, a secondary analysis of up to three cycles in the ESTHER-1 and -2 trials showed that individualized follitropin delta significantly reduced moderate/severe OHSS and/or preventive interventions compared with conventional follitropin alfa. The greatest benefit was observed in participants with AMH ≥25.35 pmol/l (highest quartile), where 11.8% of participants who received individualized follitropin delta experienced moderate/severe OHSS and/or preventive interventions compared with 22.1% of participants who received conventional follitropin alfa dosing (odds ratio, 0.47; 95% CI: 0.26; 0.86; *P *=* *0.012) (Fernández-Sánchez *et al.*, 2019).

### Limitations

There are some limitations on how the data from the BEYOND trial can be interpreted. Even if we expect a consistent follitropin delta efficacy and safety profile across different populations (as discussed above), the BEYOND trial included only European patients. As the BEYOND trial was exploratory, the data only show that using individualized follitropin delta dosing is equally effective and safe when used in either a GnRH agonist or an antagonist protocol in women aged 18–40 years and AMH ≤35 pmol/l in terms of ovarian responses. The trial was not powered to detect differences in other efficacy outcomes (i.e. ongoing pregnancy rates and live births). In addition, as participants with AMH >35 pmol/l were excluded from the BEYOND trial, clinicians should remain cautious when using a GnRH agonist protocol in patients with AMH >35 pmol/l due to a greater risk of excessive ovarian response and OHSS. Although fewer than 20% of participants had an AMH level between 25 and 35 pmol/l in the BEYOND trial, a lower AMH limit of >23 pmol/l can be used to identify women with polycystic ovarian morphology to support a PCOS diagnosis (Dietz de Loos *et al.*, 2021). Nevertheless, we do not know the OHSS risk among BEYOND trial participants with AMH 23–35 pmol/l as we did not prespecify a subanalysis of participants with baseline AMH 23–35 pmol/l, or those diagnosed with PCOS.

The double blastocyst transfer procedure is important to consider as it impacts neonatal health, which is dependent on singleton/twin status. Double blastocyst transfers were permitted for participants ≥38 years with no good-quality blastocysts. In the BEYOND trial, one set of twins was born due to a double blastocyst transfer. All the other twins born resulted from a single blastocyst transfer.

It is currently standard practice to use a GnRH agonist trigger and a ‘freeze-all’ strategy as a preventive measure for high-responders at risk of OHSS; however, the BEYOND trial did not permit this as it would have confounded the primary outcome results, as discussed above. Although the outcomes of transfers with cryopreserved blastocysts were not followed up for the BEYOND trial and the cumulative live birth rates and neonatal outcomes after cryotransfer are not known, clinicians could consider using a GnRH agonist trigger for potential high responders to maximize the chances of pregnancy and reduce the risk of OHSS by using a cryopreserved transfer.

### Overall conclusions

In women with AMH ≤35 pmol/l, a fixed daily dose of follitropin delta (individualized according to body weight and AMH) resulted in a significantly higher number of oocytes retrieved when used as part of a GnRH agonist protocol compared with an antagonist protocol (estimated mean difference 1.3), and ongoing pregnancies and live births after the first fresh transfer cycle were numerically 7% higher in agonist versus antagonist protocols (though not statistically significant). Moreover, no additional safety signals were observed and there was no additional risk of OHSS when individualized doses of follitropin delta was used as part of a GnRH agonist protocol versus a GnRH antagonist protocol. The safety profile of individualized follitropin delta dosing was similar in the GnRH agonist and antagonist protocols. Neonatal health outcomes were not affected by ovarian stimulation with follitropin delta.

## Supplementary Material

deae092_Supplementary_Data_File_S1

deae092_Supplementary_Table_S1

deae092_Supplementary_Table_S2

## Data Availability

The data underlying this article will be shared on reasonable request to the corresponding author.
