# Supplementary material for: BEYOND: a randomized controlled trial comparing efficacy and safety of individualized follitropin delta dosing in a GnRH agonist versus antagonist protocol during the first ovarian stimulation cycle
Source: Hum Reprod. 2024 May 9;39(7):1481–94. doi: 10.1093/humrep/deae092 (PMC11759129; doi:10.1093/humrep/deae092)
Supplement: deae092_Supplementary_Data_File_S1 [file deae092_supplementary_data_file_s1.pdf]

Supplementary Data File S1: Multiple imputation method for primary endpoint (number of oocytes retrieved) for the BEYOND trial

- Step 1: a negative binomial regression model with age stratum and anti-Müllerian hormone (AMH) at screening as factors was fitted to data from subjects who started stimulation with follitropin delta for each GnRH analogue protocol separately.

- Step 2: 1000 complete datasets were constructed. For the construction of each complete dataset, a set of parameter values was sampled from a normal distribution with characteristics determined by the estimated parameters and covariance matrix for the two models in the first step. Missing individual subject data were then imputed from a negative binomial distribution with the sampled parameters.

- Step 3: each complete dataset was analysed using the negative binomial regression model described in the first paragraph of this section.

- Step 4: the estimated mean differences and the standard deviations from the 1000 analyses were combined using Rubin's formula:

$$m_{MI} = \frac{1}{1000}\sum_{i=1}^{1000} m_i, \quad SD_{MI} = \sqrt{\frac{1}{1000}\sum_{i=1}^{1000} {SD_i}^2 + \left(1+\frac{1}{1000}\right)\left(\frac{1}{1000-1}\right)\sum_{i=1}^{1000}(m_i - m_{MI})^2}$$

Where m_i and SD_i were the estimated mean differences and standard deviations for the differences for the 1000 copies of the dataset (derived using the delta method), and m_{MI} and SD_{MI} were the pooled estimates.

- The mean difference in number of oocytes retrieved between the gonadotropin-releasing hormone (GnRH) agonist protocol and the GnRH antagonist protocol was estimated as m_{MI}, and the 95% CI for the estimate was calculated as $m_{MI} \pm 1.96 * SD_{MI}$.

The assumption underlying the above imputation procedure was that all randomised subjects could have started stimulation with follitropin delta, and that their outcome would not have differed in any systematic way from the subjects who started stimulation with follitropin delta.

For subjects who discontinued the trial after start of stimulation with follitropin delta but before oocyte retrieval the number of oocytes retrieved was regarded as zero.

Supplementary analyses and descriptions for the primary endpoint

The primary analysis was repeated based on subjects in full analysis set who started stimulation with follitropin delta, i.e., without using multiple imputations (this was the same population as the safety analysis set.)

The potential influence of centre differences on the results for the primary endpoint was investigated by repeating the analyses of the 1000 complete datasets generated above, with the addition of centre and the interaction between centre and age strata as factors in the model.

Additional analyses were made for two subgroups based on AMH at screening (<15 pmol/L or ≥15 pmol/L) and for the three age strata (<35, 35–37, or 38–40 years). These analyses were based on the 1000 complete datasets used for the primary analyses and used the same analysis model, except that age stratum was not included as factor in the age strata analyses and AMH was not included in the AMH subgroup analyses. The results of the subgroup analyses were illustrated in a forest plot.

The distribution of number of oocytes retrieved was described using the empirical distribution and approximated using kernel estimates.
