# Supplementary material for: BEYOND: a randomized controlled trial comparing efficacy and safety of individualized follitropin delta dosing in a GnRH agonist versus antagonist protocol during the first ovarian stimulation cycle
Source: Hum Reprod. 2024 May 9;39(7):1481–94. doi: 10.1093/humrep/deae092 (PMC11759129; doi:10.1093/humrep/deae092)
Supplement: deae092_Supplementary_Table_S1 [file deae092_supplementary_table_s1.pdf]

OXFORD
human reproduction

Human Reproduction, 2024, 1–1
https://doi.org/10.1093/humrep/deae092
Supplementary Data

Supplementary Table S1. Inclusion and exclusion criteria for BEYOND trial.

Inclusion criteria	Exclusion criteria
Subjects had to meet *all* of the criteria listed below to be eligible for participation in the trial.<br><br>1. Informed Consent Forms signed prior to screening evaluations.<br>2. In good physical and mental health.<br>3. The subjects must be at least 18 years (including the 18th birthday) when they sign the Informed Consent Form and no more than 40 years (up to the day before the 41st birthday) at the time of randomization.<br>4. Infertile women diagnosed with tubal infertility, unexplained infertility, endometriosis stage I/II or with partners diagnosed with male factor infertility, eligible for IVF and/or ICSI using fresh or frozen ejaculated sperm from male partner or sperm donor.<br>5. Infertility for at least 1 year before randomization for subjects <38 years or for at least 6 months for subjects ≥38 years (not applicable in case of tubal or severe male factor infertility).<br>6. The trial cycle will be the subject's first controlled ovarian stimulation cycle for IVF/ICSI.<br>7. Regular menstrual cycles of 24–35 days (both inclusive), presumed to be ovulatory.<br>8. Hysterosalpingography, hysteroscopy, saline infusion sonography, or transvaginal ultrasound documenting a uterus consistent with expected normal function (e.g. no evidence of clinically interfering uterine fibroids defined as submucous or intramural fibroids larger than 3 cm in diameter, no polyps and no congenital structural abnormalities which are associated with a reduced chance of pregnancy) within 1 year prior to randomization.<br>9. Transvaginal ultrasound documenting presence and adequate visualization of both ovaries, without evidence of significant abnormality (e.g. no endometrioma greater than 2 cm or enlarged ovaries which would contraindicate the use of gonadotropins) and normal adnexa (e.g. no hydrosalpinx) within 1 year prior to randomization. Both ovaries must be accessible for oocyte retrieval.<br>10. Early follicular phase (Cycle Days 2–5) serum levels of FSH between 1 and 15 IU/l at screening.<br>11. Negative serum hepatitis B surface antigen, hepatitis C virus, and HIV antibody tests within 1 year prior to randomization.<br>12. BMI between 17.5 and 32.0 kg/m^2 (both inclusive) at screening.<br>13. If <38 years willing to accept single blastocyst transfer. If ≥38 years willing to accept transfer of a single good-quality blastocyst (double blastocyst transfer may be performed if no good-quality blastocyst is available).	Subjects who met any of the criteria listed below were *not* eligible for participation in the trial.<br><br>1. AMH >35 pmol/l at screening.<br>2. Strong preference of the subject for either treatment protocol.<br>3. Known endometriosis stages III–IV (defined by the revised ASRM classification, 1996).<br>4. Known history of recurrent miscarriage (defined as three consecutive losses after ultrasound confirmation of pregnancy (excl. ectopic pregnancy) and before week 24 of pregnancy).<br>5. Known abnormal karyotype of subject or of her partner/sperm donor, as applicable, depending on source of sperm used for insemination in this trial.<br>6. Any known clinically significant systemic disease (e.g. insulin-dependent diabetes).<br>7. Known inherited or acquired thrombophilia disease.<br>8. Active arterial or venous thromboembolism or severe thrombophlebitis, or a history of these events.<br>9. Known porphyria.<br>10. Any known endocrine or metabolic abnormalities (pituitary, adrenal, pancreas, liver, or kidney) with the exception of controlled thyroid function disease.<br>11. Known tumours of the ovary, breast, uterus, adrenal gland, pituitary, or hypothalamus which would contraindicate the use of gonadotropins.<br>12. Known moderate or severe impairment of renal or hepatic function.<br>13. Currently breast-feeding.<br>14. Undiagnosed vaginal bleeding.<br>15. Known abnormal cervical cytology of clinical significance observed within 3 years prior to randomization (unless the clinical significance has been resolved).<br>16. Findings at the gynaecological examination at screening which preclude gonadotropin stimulation or are associated with a reduced chance of pregnancy, e.g. congenital uterine abnormalities or retained intrauterine device.<br>17. Pregnancy (negative pregnancy test must be documented at screening) or contraindication to pregnancy.<br>18. Known current active pelvic inflammatory disease.<br>19. Use of fertility modifiers during the last menstrual cycle before randomization, including dehydroepiandrosterone, metformin or cycle programming with oral contraceptives, progestogen or oestrogen preparations.<br>20. Use of hormonal preparations (except for thyroid medication) during the last menstrual cycle before randomization.<br>21. Known history of chemotherapy (except for gestational conditions) or radiotherapy.<br>22. Current or past (1 year prior to randomization) abuse of alcohol or drugs and/or current (last month) intake of more than 14 units of alcohol per week.<br>23. Current or past (3 months prior to randomization) smoking habit of more than 10 cigarettes per day.<br>24. Hypersensitivity to any active ingredient or excipients in the medicinal products used in the trial.<br>25. Previous participation in the trial.<br>26. Use of any nonregistered investigational drugs during the last 3 months prior to randomization.
