# Supplementary material for: BEYOND: a randomized controlled trial comparing efficacy and safety of individualized follitropin delta dosing in a GnRH agonist versus antagonist protocol during the first ovarian stimulation cycle
Source: Hum Reprod. 2024 May 9;39(7):1481–94. doi: 10.1093/humrep/deae092 (PMC11759129; doi:10.1093/humrep/deae092)
Supplement: deae092_Supplementary_Table_S2 [file deae092_supplementary_table_s2.pdf]

OXFORD

human reproduction

Human Reproduction, 2024, 1–1
https://doi.org/10.1093/humrep/deae092
Supplementary Data

Supplementary Table S2. Ovarian response and ongoing pregnancies stratified by age (safety analysis set).

	GnRH agonist protocol (n = 202)	GnRH antagonist protocol (n = 204)	Difference (95% CI) and *P-value*
<35 years	n = 130	n = 131	–
Oocytes retrieved (multiple imputations)	12.0 ± 6.1	10.2 ± 5.8	1.52 (0.05; 2.99); **P = 0.0431**[a]
Poor responders (<4 oocytes)	6 (4.6)	16 (12.2)	–
Excessive responders (≥15 oocytes)	43 (33.1)	31 (23.6)	–
Excessive responders (≥20 oocytes)	13 (10.0)	10 (7.6)	–
Ongoing pregnancy	53 (40.8)	40 (30.5)	–
Singleton	50	39	–
Twin	3	1	
35–37 years	n = 41	n = 47	–
Oocytes retrieved (multiple imputations)	9.8 ± 4.5	8.6 ± 4.3	1.19 (−0.67; 3.05); P = 0.210[a]
Poor responders (<4 oocytes)	2 (4.9)	5 (10.6)	–
Excessive responders (≥15 oocytes)	8 (19.5)	5 (10.6)	–
Excessive responders (≥20 oocytes)	0	0	–
Ongoing pregnancy (as observed)	13 (31.7)	14 (29.8)	–
Singleton	13	14	–
Twin	0	0	
38–40 years	n = 31	n = 26	
Oocytes retrieved (multiple imputations)	9.4 ± 6.4	7.5 ± 8.4	0.54 (−2.15; 3.23); P = 0.6926[a]
Poor responders (<4 oocytes)	2 (4.9)	5 (10.6)	–
Excessive responders (≥15 oocytes)	8 (19.5)	5 (10.6)	–
Excessive responders (≥20 oocytes)	0	0	–
Ongoing pregnancy	7 (22.6)	6 (23.1)	–
Singleton	6	6	–
Twin	1	0	–

[a] Treatment group comparative analyses by subgroup were based on all randomized subjects using a multiple imputation method for subjects withdrawing before the start of stimulation (GnRH agonist group: <35 years, n = 143; 35–37 years, n = 46; 38–40 years, n = 31; GnRH antagonist group: <35 years, n = 139; 35–37 years, n = 49; 38–40 years, n = 27). *P*-values in bold text are less than 0.05 (i.e., statistically significant).
CI, confidence interval; GnRH, gonadotropin-releasing hormone.
